# Tunable Fluorescence of Graphdiyne Quantum Dots by Enlarging π System and Altering Electronic Structure

**DOI:** 10.3390/molecules31213998

**Published:** 2026-11-06

**Authors:** Ling Qiao, Wenchao Lang, Jia Luo, Xiaoqing Xu

**Affiliations:** 1 School of Pharmacy and Laboratory Medicine, Jiangsu Health Vocational College, Nanjing 211800, China; qiaoling0516@126.com (L.Q.);; 2 School of Chemistry, Chemical Engineering & Biotechnology, Nanyang Technological University, Singapore 637371, Singapore

**Keywords:** graphdiyne, quantum dots, multicolor, fluorescence, bioimaging

## Abstract

Extending the fluorescence of graphdiyne quantum-dot-derived materials beyond the predominantly reported blue-to-green region may broaden their use in fluorescence imaging and sensing. To address this issue, modified graphdiyne quantum dots (M-GDQDs) with green, yellow, and red fluorescence were first obtained by classical hydrothermal and solvothermal methods. Experimental and theoretical studies have shown that the enlargement of the π system and the changes in electronic structures contributed to the red shift in the fluorescence emission wavelength, although the individual contributions of conjugation and surface chemistry could not be quantitatively separated. The prepared M-GDQDs exhibit wonderful optical properties, good water solubility and excellent biocompatibility, which are beneficial for bioimaging. This study not only enriches the types of graphdiyne quantum dots, but it can also be used for the construction of multichannel fluorescent biosensing platforms. We believe that the emergence of M-GDQDs offers promising application prospects in the fields of nanomedicine and disease-related biomarker analysis.

## 1. Introduction

The existence of graphynes (GYs) was theoretically predicted by Baughman et al. in 1987, which are a new type of 2D carbon-based materials formed by both sp and sp^2^ hybridized carbon atoms according to some periodic rule [1]. And graphdiyne (GDY) is the first graphyne obtained from experiment by Li et al. in 2010 [2], which is formed by fundamental structural unit with big triangular ring containing 18 carbon atoms, and there are two acetenyl groups (C≡C) between benzene rings. Having benefited from the novel carbon allotrope and its distinct properties including uniformly distributed pores, high π conjugations, electrical, optical, and mechanical properties, GDY and its derivatives have drawn tremendous attention in the fields of catalysis, energy, and environment [3,4,5,6]. In addition, GDY was first applied in fluorescent biosensing in 2016, and then Jia et al. reported that GDY oxide (GDYO) is more stable in biological system than graphene oxide (GO) and possesses less biotoxicity to cellular activity [7,8]. Therefore, GDY-based nanomaterials are now being used for biosensing, antibacterial, anticancer, and drug delivery studies owing to its excellent biocompatibility and stability. And there are some critical reviews that established biomedical applications of GDY-based materials [9,10].

For further extending the applications of GDY, graphdiyne quantum dots (GDQDs), a novel graphdiyne derivative with perfect crystallization and uniform particle size, have attracted a considerable amount of attention because of their remarkable qualities, such as low toxicity, good solubility, fluorescence, electrochemical activity, and peroxidase-like activity. For example, Liu et al. first synthesized GDQDs from GD flakes using a solvothermal approach in chlorobenzene (CB) or dimethylsulfoxide (DMSO) with the assistance of ultrasonication and magnetic stirring in 2018, and introduced GDQDs into perovskite solar cells to improve their light-harvesting capacity, reduce conduction band offset, and increase device stability [11,12]. As one of the derivatives of GDY, GDQDs inherited the excellent optical, electrical, and biocompatibility of GDY because of its uniformly distributed sub-nanopores, large π-conjugated system and active acetylene units. In 2019, Nie’s team obtained GDQDs with green fluorescence by classic hydrothermal method and explored its biosafety in vitro and in vivo for biomedical applications [13]. And next year Sui et al. first fabricated a biosensor for the simultaneous analysis of iron ions and ascorbic acid (AA) using the blue-green fluorescence of GDQDs with abundant C≡C obtained from the ultrasonic method [14]. In addition, Salimi’s laboratory designed a ratiometric fluorescent assay for sensitive detection of cancer biomarker miRNA-21. In this system, the GDQDs serve as energy donors because of their strong and steady fluorescence emission at 405 nm, and their good biocompatibility endowed them with great potential in bioimaging [15]. Taking advantage of the outstanding electrochemical properties of sp-hybridized N-doped graphdiyne, our team successfully acquired a type of nitrogen-doped graphdiyne quantum dots (NGDYQDs) through hydrothermal treatment and utilized them to develop an electrochemical sensor for the quantification of chloramphenicol (CP) [16]. Then, Sui’s lab also applied nitrogen-doped graphdiyne dots (N-GDQDs) to construct an optical-electrochemical sensor for detection of dopamine with high sensitivity and selectivity [17]. Furthermore, some reports in the literature report that graphdiyne-based nanomaterials could already be used as novel nanozymes [18,19,20], and graphdiyne oxide quantum dots (GDYO QDs) were also explored as a kind of peroxidase-like enzyme due to their size effect by Sun et al. in 2022 [21]; they studied the catalytic scheme and applied it to analysis of H_2_O_2_ and L-cysteine. At the same time, Sui et al. constructed hollow cube-shaped N-GDQDs/AuAg nanocages (AuAg NCs) with enhanced peroxidase-like activity thanks to the narrow band gap of N-GDQDs and LSPR effect of AuAg NCs, which they then used for nanozymes in antimicrobial applications [22].

Up to now, many studies about GDQDs have been reported which can be employed for energy storage, bioimaging and biosensing. However, many GDQDs described in the studies discussed above exhibit emission in the blue-to-green region, which may restrict their use in applications requiring longer-wavelength fluorescence. The present study therefore explores a series of GDQD-derived materials with emissions extending into the yellow and red regions; this distinction is made without implying that other classes of carbon-based quantum dots lack multicolor emission. In addition, it is necessary to fabricate multichannel fluorescence biosensing platform for biochemical analysis and improve the enzymatic activity of GDQDs-based nanozymes. Therefore, it is an urgent issue and a great challenge for researchers to fabricate GDQDs with multicolor fluorescence emission wavelengths, and it is also meaningful to exploit new GDQDs to expand their present functions and fulfill application requirements in various fields. And bandgap narrowing could be achieved by expanding the size of the sp^2^ domain (that is enlarging the π system) and introducing electron-donating groups (that is altering electronic structure) according to the previous literature [23,24]. Building on these observations, we investigated the fluorescence of modified GDQDs prepared with three aromatic amines, evaluated trends in their optical and electronic properties, and explored their cellular uptake and fluorescence imaging in cultured cells.

## 2. Results and Discussion

### 2.1. Synthesis and Characterization of GDQDs and M-GDQDs

**Scheme 1:**
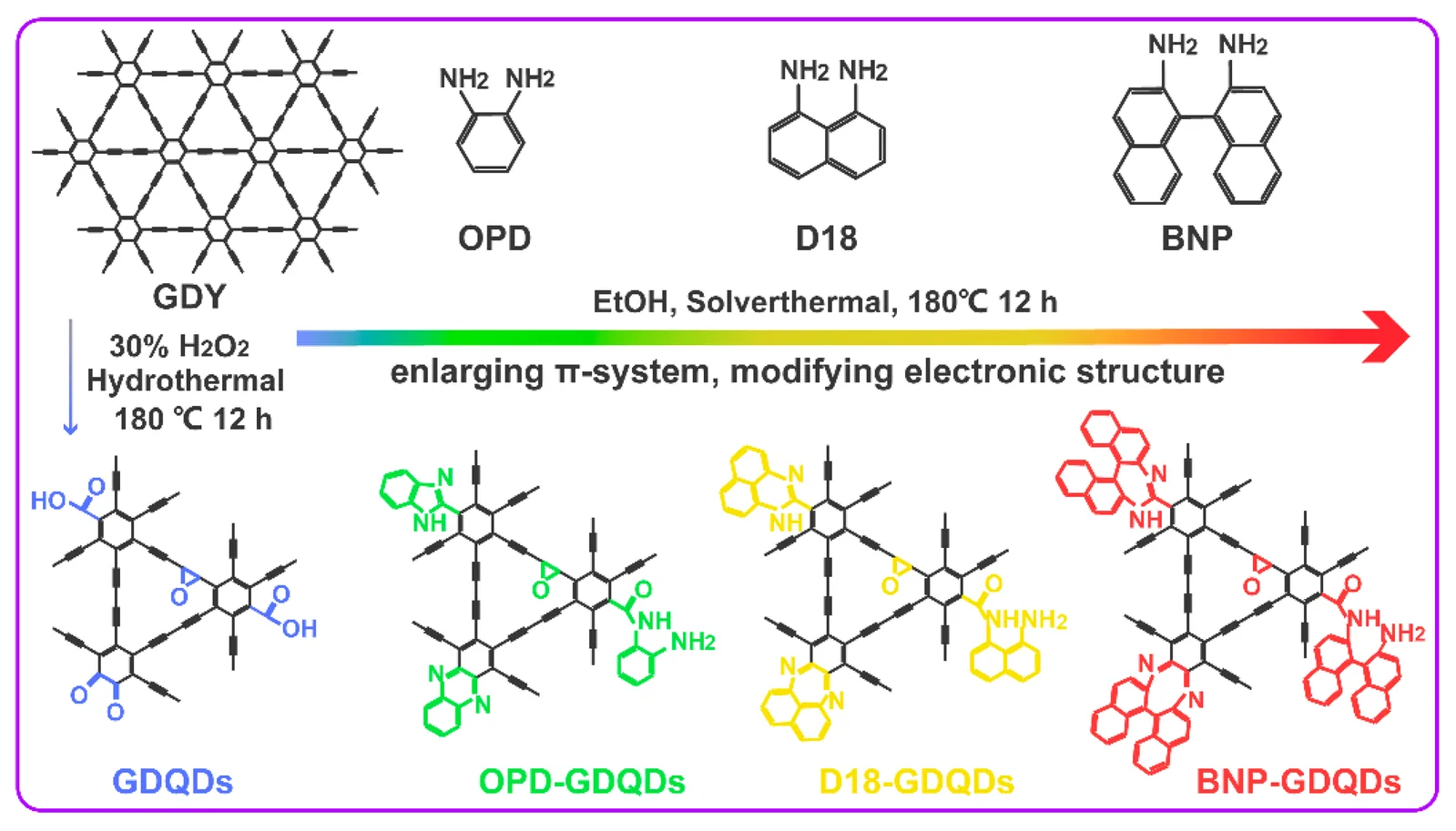
Illustration of the synthetic route of graphdiyne quantum dots with varying fluorescence. For clarity, we selected the structure of GDQDs without hydroxyl after oxidation for the explanation; the reaction results were identical whether carbonyl or hydroxyl was used.

We experimentally prepared four typical graphdiyne quantum dots with blue, green, yellow, and red fluorescence by both hydrothermal and solvothermal treatments with the aid of ultrasonicate (the preparation method is detailed in the Experimental Section). As depicted in Scheme 1, we first prepared graphdiyne quantum dots (GDQDs) with blue emission fluorescence from graphdiyne using the hydrothermal method (See Experimental Section). Then, diverse polycyclic aromatic compounds, such as *o*-phenylenediamine (OPD), 1,8-diaminonaphthalene (D18), and 1,1′-bi(2-naphthalene) (BNP) were reacted with GDQDs to generate modified graphdiyne quantum dots (M-GDQDs) with phenazine, amide, and benzimidazole structures after condensation reactions (OPD-GDQDs with green fluorescence, D18-GDQDs with yellow fluorescence, and BNP-GDQDs with red fluorescence). Lastly, we employed the GDQDs and M-GDQDs for FL bioimaging in MCF-7 cells thanks to their brilliant fluorescence properties and excellent biosafety. To the best of our knowledge, this is the first time that multicolor luminescent graphdiyne quantum dots have been reported.

**Figure 1:**
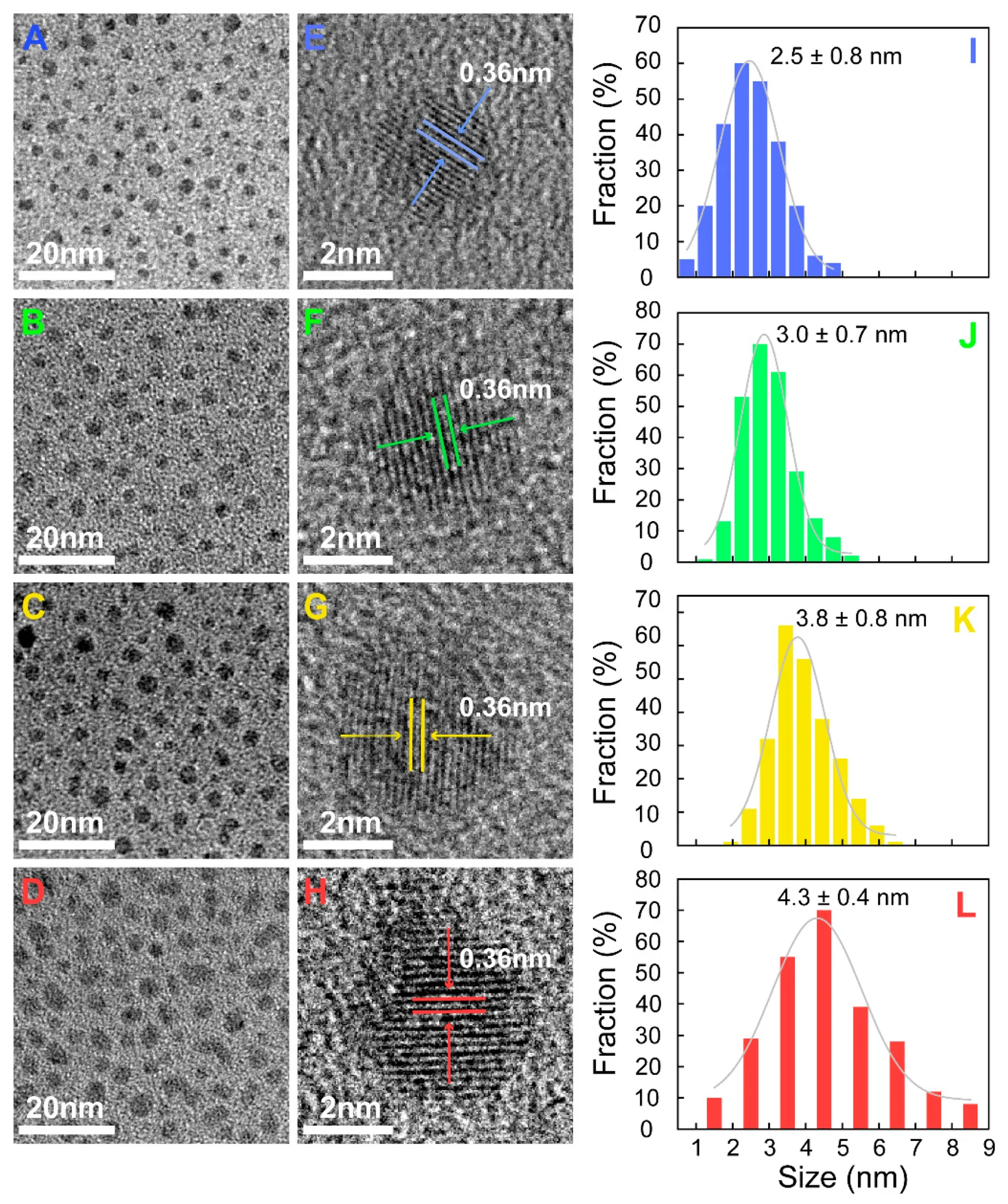
TEM images (**A**–**D**), HRTEM images (**E**–**H**), and size distributions (**I**–**L**) of the synthesized GDQDs, OPD-GDQDs, D18-GDQDs, and BNP-GDQDs.

Transmission electron microscopy (TEM) images of the four quantum dots are shown in Figure 1A–D, revealing that all quantum dots are spherical particles with uniform distribution. HRTEM images display that all of them have identical visible crystalline structure with a lattice fringe space of 0.36 nm (Figure 1E–H), which corresponds to the 002 plane of graphite and is consistent with the previous literature [25]. As depicted in Figure 1I–L, the average particle size of GDQDs, OPD-GDQDs, D18-GDQDs, and BNP-GDQDs is determined by randomly selecting 150 particles, and corresponding values are 2.5 ± 0.8, 3.0 ± 0.7, 3.8 ± 0.8, and 4.3 ± 0.4 nm, respectively. In addition to demonstrating that GDQDs and M-GDQDs have an excellent crystalline structure, the results indicate that the mean particle size exhibits a slight upward trend from blue GDQDs to red BNP-GDQDs.

As observed from Figure S1, the X-ray diffraction (XRD) pattern of GDQDs displays a broad peak at 22–26°, which is attributed to the (002) diffraction peak of graphite, and its broadness is attributed to the small particle size and defects in the crystal structure after hydrothermal treatment [26,27]. The results also indicate that the peaks are becoming sharper from GDQDs to BNP-GDQDs, possibly due to the effective conjugation of GDQDs with polycyclic aromatic compounds. In addition, FWHMs (Full Width at Half Maximum) of GDQDs, OPD-GDQDs, D18-GDQDs, and BNP-GDQDs are 15.93°, 14.44°, 7.71°, 7.63°, respectively. Mild enhancement of relative intensity of the diffraction peaks from the blue line to the red line also suggests that BNP-GDQDs retain a longer effective conjugation length than the other GDQDs, with GDQDs being the shortest [28]. From the results of TEM and XRD, we can conclude that the crystallinity and effective conjugation length of M-GDQDs improves while GDQDs are subjected to extra solvothermal treatment and form a conjugating structure with OPD, D18, and BNP. Moreover, we speculate it may be beneficial to the red shift of emission wavelength from blue GDQDs to red BNP-GDQDs.

**Figure 2:**
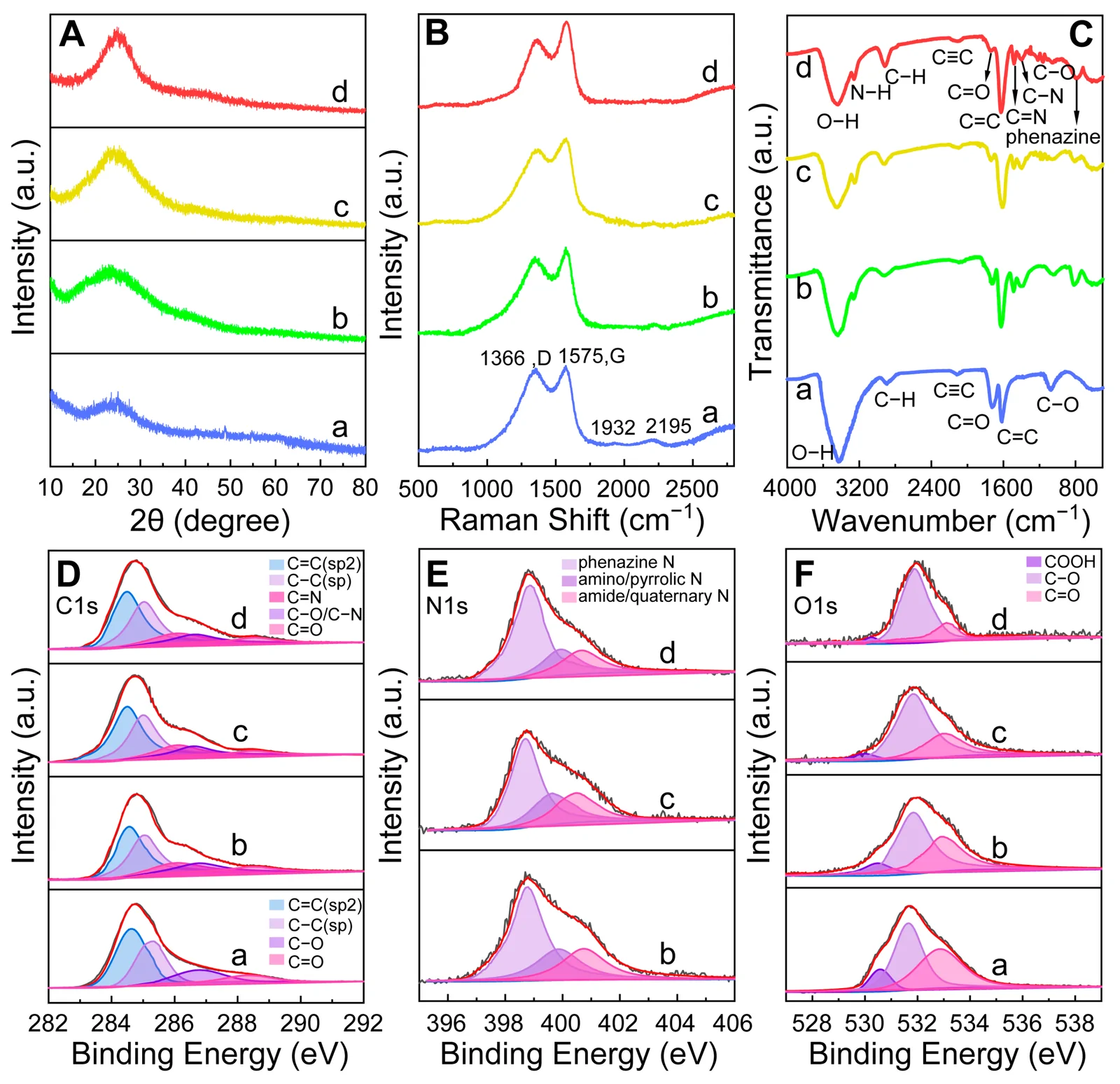
Characterizations of the four quantum dots. (**A**) Raman spectra, (**B**) FTIR spectra, and (**C**) XPS survey of GDQDs (blue line a), OPD-GDQDs (green line b), D18-GDQDs (yellow line c), and BNP-GDQDs (red line d). (**D**–**F**) High-resolution spectra of C 1 s, N 1 s, and O 1 s. Characters a, b, c, and d respectively represent GDQDs, OPD-GDQDs, D18-GDQDs, and BNP-GDQDs.

In addition to TEM and XRD, Raman spectra were used to ascertain the enhancement of crystallinity and effective conjugation length. Figure 2A displays two prominent peaks at 1366 cm^−1^ and 1575 cm^−1^, which correspond to the disordered defect structure D band and crystalline structure G band of graphdiyne, respectively. From line a to line d, the intensity of the G band increase noticeably and two minor peaks at 1932 cm^−1^ and 2195 cm^−1^ become progressively weaker [2,16]. However, there is no discernible difference in the D band between GDQDs and M-GDQDs; the I_D_/I_G_ values for GDQDs, OPD-GDQDs, D18-GDQDs, and BNP-GDQDs are listed in Table S1.

Next, Fourier transform infrared spectroscopy (FTIR) and X-ray photoelectron spectroscopy (XPS) analyses were utilized to further elucidate the formation of conjugating structures. Figure 2B verifies that all quantum dots have characteristic peaks at 3435 cm^−1^, 3067 cm^−1^, 2100 cm^−1^, 1690 cm^−1^, 1616 cm^−1^, and 1080 cm^−1^, which correspond to the stretching vibrations of O-H, sp^2^ C-H, C≡C, C=O, C=C, and C-O, respectively. Meanwhile, the three M-GDQDs exhibit absorption bands at approximately 3260 cm^−1^ (N–H stretching), 1530 cm^−1^ (C=N stretching), and 1375 cm^−1^ (C–N vibrations) [12,29,30,31]. And 750 cm^−1^ may be associated with skeletal vibrations of phenazine-related structures, consistent with the FTIR results reported by Tetsuka et al. [32].

Both the distinct drop in intensity of oxygen-containing groups and significant increase in intensity of the C=C band derived from the sp^2^ C framework imply that the condensation reaction was successful, while the tiny change in the intensity of the C≡C peak demonstrates that three kinds of modified quantum dots retain the graphdiyne structure of GDQDs after being exposed to a harsh environment. Notably, the appearance of nitrogen-containing functional groups and phenazine structures indicates that the electronic structures of M-GDQDs after the condensation reaction differ from GDQDs, and this discrepancy is likely to be favorable for the red shift in the emission peak position because the electron-withdrawing ability of nitrogen-containing groups can narrow the HOMO (highest occupied molecular orbital)-LUMO (lowest unoccupied molecular orbital) band gap [33], and the reason for the red shift of fluorescence emission wavelength.

XPS measurement was performed here to analyze the chemical composition and element content of quantum dots. Figure 2C exhibits the full scan XPS spectrum of GDQDs and M-GDQDs, the peaks at 284.8 eV and 532.1 eV represent C 1 s and O 1 s of all quantum dots, respectively, and the peak appears at 401.1 eV in line b-d is the peculiar band of N 1 s. As seen in Table S2, the C and O content tend to rise and decline from GDQDs to M-GDQDs, respectively, which is consistent with the FTIR results, whereas the nitrogen content lessens slightly, resulting in a boost in the C 1 s/O 1 s atomic ratio and a reduction in the N 1 s/C 1 s atomic ratio (Table S3). Figure 2D–F portray the high-resolution XPS spectra for C 1 s, N 1 s, and O 1 s, respectively. For M-GDQDs (line b-d in Figure 2D), the C 1 s envelope can be fitted into five peaks: C=C (sp^2^ C, 284.53 eV), C≡C (sp C, 285.02 eV), C=N (286.07 eV), C-O/C-N (286.60 eV), and C=O (288.50 eV). However, for blue GDQDs (line a), it only contains four peaks at 284.53 eV, 285.02 eV, 286.60 eV, 288.50 eV for C=C, C≡C, C-O, C=O [34,35]. Table S4 records the composition of various carbon bonding calculated from C 1 s XPS, and the integrated area ratios of sp^2^ C/sp C for GDQDs, OPD-GDQDs, D18-GDQDs, and BNP-GDQDs are 1.06, 1.25, 1.30, and 1.45, respectively (Table S5). It is suggested that the addition of polycyclic aromatic amines after solvothermal led to the π system enlargement of the M-GDQDs and retained the graphdiyne structure. The N 1 s spectrum (Figure 2E) of all M-GDQDs consists of three component bands: phenazine N located at 398.7 eV resulting from the reaction between carbonyl/hydroxyl and aromatic amides; amino/pyrrolic N at 399.8 eV attributable to the amide and benzimidazole structures formed by the reaction of the carboxyl with three different chemicals; and amide/quaternary N situated at 400.6 eV originating from the condensation reaction of carboxyl groups with OPD, D18, BNP [24,36,37]. The percentage contents of N 1 s-relevant chemical bonds are listed in Table S6; in view of the fact that the amino N and amide N resulting from the amide structures, and the pyrrolic N and quaternary N present in the benzimidazole skeletons, always appear in pairs, the chemical bond represented by 399.8 eV and 400.6 eV is nearly equivalent. In addition, the O 1 s XPS for GDQDs and M-GDQDs can be converted into three Gaussian peaks centered at 530.1 eV, 531.8 eV, and 533.0 eV, which correspond to COOH, C-O, C=O, respectively (Figure 2F) [13]. From line a to line d, the contents of COOH groups and C=O groups are reduced drastically, which is quantified in Table S7.

Together, these results support changes in the surface chemical environment and carbon bonding after the modification process. The increasing sp^2^ C/sp C area ratio (1.06, 1.25, 1.30 and 1.45) accompanies the progressive emission shift (465, 540, 600 and 650 nm). This association is consistent with an important role of extended conjugation. However, nitrogen-related electronic effects, surface states, structural heterogeneity and particle-size changes may also influence the optical response; these effects are not independently varied in the present work.

The synthesized GDQDs and M-GDQDs were subjected to repeated centrifugation and washing, followed by dialysis using a membrane with a molecular weight cutoff of 500 Da for three days and subsequent freeze-drying. These purification procedures were intended to reduce the presence of unreacted precursors and freely diffusible low-molecular-weight byproducts. The molecular weights of OPD, D18, and BNP are 108.14, 158.20, and 284.36 g/mol, respectively, all below the nominal dialysis cutoff.

Nevertheless, we acknowledge that these purification procedures cannot conclusively eliminate all molecular fluorophores, particularly those associated with the nanoparticle surfaces or retained through aggregation and intermolecular interactions. The TEM and HRTEM observations confirm the presence of nanoscale carbonaceous structures, while the FTIR, XPS, and Raman results provide evidence of changes in the chemical bonding environments following aromatic amine treatment. However, these observations alone cannot unambiguously distinguish carbon-core emission from nanoparticle-associated molecular-state emission or exclude contributions from residual free molecular fluorophores. Therefore, the observed fluorescence is discussed in terms of possible contributions from the modified conjugated structures and their associated emissive states, while the precise contributions of different fluorescent species remain to be further elucidated.

### 2.2. Photoluminescence Properties of GDQDs and M-GDQDs

**Figure 3:**
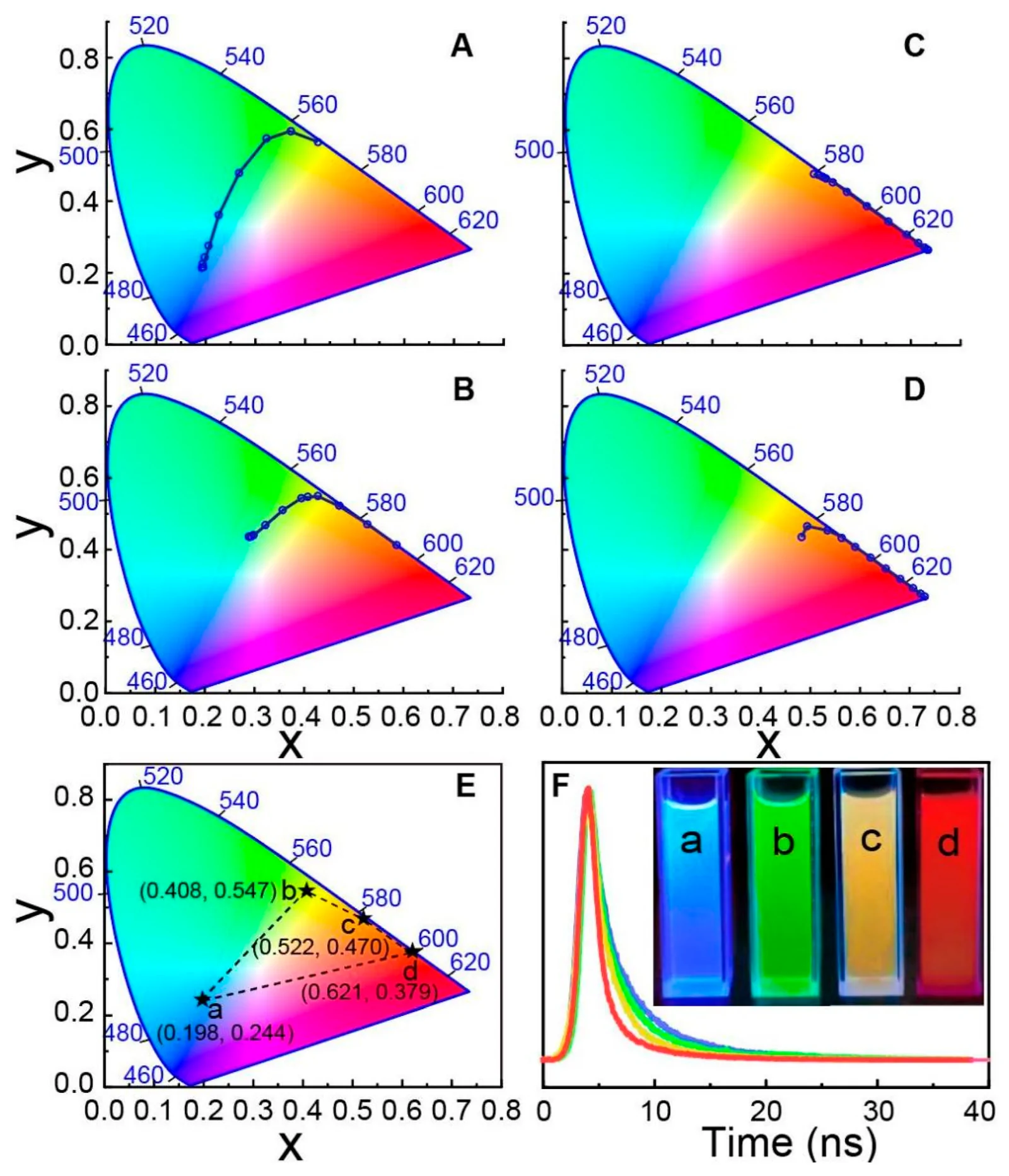
CIE 1931 color coordinates for GDQDs (**A**), OPD-GDQDs (**B**), D18-GDQDs (**C**), and BNP-GDQDs (**D**) under different excitation wavelengths. © CIE 1931 coordinates of maximum PL emission spectra. (**E**) CIE 1931 coordinates of maximum PL emission spectra. (**F**) PL decay of GDQDs (blue line), OPD-GDQDs (green line), D18-GDQDs (yellow line), and BNP-GDQDs (red line) under different conditions, the inset shows fluorescence images of four typical quantum dots illuminated by a 365 nm UV lamp. Character a, b, c, and d represent GDQDs, OPD-GDQDs, D18-GDQDs, and BNP-GDQDs, respectively.

PL emission and excitation spectra were first applied to illustrate the photoluminescence properties of the synthetic quantum dots. Figure S2A–D represent the fluorescence emission spectra of GDQDs and M-GDQDs at different excitation wavelengths, revealing that all kinds of quantum dots show an excitation dependence, with maximum fluorescence emission at 465 nm, 540 nm, 600 nm, and 650 nm, respectively. And their CIE 1931 coordinates under various excitation wavelengths are presented in Figure 3A–D. Intuitively, the results reveal that the emission wavelengths of the four quantum dots exhibit a red shift from the blue to red light region, which is similar to the aforementioned increase in particle size due to the enlargement of the π system, indicating that enlarging the π system can indeed regulate the fluorescence properties of graphdiyne quantum dots. Figure 3E shows the CIE 1931 color coordinates of various quantum dots calculated from the normalized PL spectrum in Figure S3 (indicates that the maximum emission wavelength shifts gradually from 465 nm to 650 nm with the change in excitation wavelengths from the blue line a to the red line d). The CIE 1931 coordinates for GDQDs, OPD-GDQDs, D18-GDQDs, and BNP-GDQDs appeared at (0.198, 0.244), (0.408, 0.547), (0.522, 0.470), and (0.621, 0.379), respectively, the result reconfirmed the fact of a vivid PL red shift. Then, the maximum PLE excitation spectra of four typical quantum dots are shown in Figure S4A–D. The results indicate that they possess nonidentical PLE behavior; for GDQDs, the PLE spectra are concentrated in the ultraviolet region, while for OPD-GDQDs, D18-GDQDs, and BNP-GDQDs, the PLE spectra are shifted from ultraviolet to visible region. And their optimal excitation wavelengths are located at 380 nm, 467 nm, 471 nm and 530 nm, respectively, which also shows a gradual red-shifting trend with the increasing π system. The excitation-dependent emission is also consistent with the possibility of heterogeneous emissive environments, including distinct surface states or conjugated domains. The proposed structural motifs should therefore be understood as illustrative rather than a unique description of every emitting center.

Thirdly, time-resolved photoluminescence (PL) measurements were further performed to investigate the fluorescence decay behavior of GDQDs and M-GDQDs. As shown in Figure 3F, the fluorescence decay curves of all four quantum dots were fitted using a biexponential model under their respective optimal excitation wavelengths, and the inset exhibited the corresponding optical images of GDQDs, OPD-GDQDs, D18-GDQDs, and BNP-GDQDs under 365 nm UV lamp irradiation. The corresponding average lifetime (τ_Av_) were listed in Table S8. The τ_Av_ values of GDQDs, OPD-GDQDs, D18-GDQDs, and BNP-GDQDs were 2.57, 2.50, 1.95, and 1.23 ns, respectively, exhibiting a decreasing trend with the fluorescence red shift [38]. Meanwhile, the absolute fluorescence quantum yields (QYs), determined using an integrating sphere, decreased progressively from 14.96% for GDQDs to 13.57%, 10.01%, and 7.41% for OPD-GDQDs, D18-GDQDs, and BNP-GDQDs, respectively. Similar to τ_Av_, the result shows a downward trend of QYs when their PL emission is red-shifted [39]. Meanwhile, the lower QYs may be relevant to the introduction of electron-withdrawing groups after condensation, and it also implies that the dissimilar fluorescence arises from the final conjugates as opposed to polycyclic aromatic compounds [40,41,42]. To gain further insight into the decrease in QYs, the effective radiative decay rates (*k*_r)_ and non-radiative decay rates (*k*_nr_) were estimated from the absolute QYs(Φ) and average fluorescence lifetimes (τ_Av_) using the relationships *k*_r_ = Φ/τ_Av_ and *k*_nr_ = (1 − Φ)/τ_Av_. As summarized in Table S8, the estimated *k*_r_ values were 0.0582, 0.0543, 0.0513, and 0.0602 ns^−1^, whereas the corresponding *k*_nr_ values were 0.3309, 0.3457, 0.4615, and 0.7528 ns^−1^, respectively. Notably, *k*_nr_ increased progressively with the emission redshift, while *k*_r_ showed no consistent decreasing trend. These findings suggest that the increasing contribution of non-radiative relaxation pathways plays an important role in the reduction in fluorescence QYs. The enhanced non-radiative relaxation may be associated with changes in the electronic structure, surface functional groups, and local emissive environments following the modification of GDQDs. Nevertheless, the relative contributions of these factors cannot be independently determined from the present data. Overall, these results provide further insight into the fluorescence behavior of M-GDQDs and suggest that the observed changes in fluorescence efficiency are associated with the combined effects of structural modification and non-radiative relaxation processes.

Lastly, the fluorescence stability of quantum dots under various pH, ionic strengths, and excited times was evaluated. Figure S5 exemplifies that these four quantum dots fluctuate slightly within the neutral range and decline in acidic or alkaline solutions. In addition, the intensities of PL emission remain constant for all quantum dots at different concentrations of NaCl (0~2 mol/L), elucidating that the quantum dots’ fluorescence properties remain stable when exposed to high ionic strength (Figure S6). As elucidated in Figure S7, the four types of quantum dots retained approximately 85–90% of their initial fluorescence intensities after 120 min of irradiation by a Xenon light (150 w), which is crucial for practical applications such as imaging in living cells and in vivo. For comparison, Dager et al. reported that carbon quantum dots synthesized from fennel seeds exhibited negligible fluorescence attenuation after 4 h of continuous irradiation under a 150 W xenon lamp [43]. Wang et al. reported that carbon dots prepared from glucose and glutathione retained approximately 92% of their initial fluorescence intensity after 120 min of irradiation using a 450 W xenon lamp [44]. Although the fluorescence attenuation of the present GDQDs and M-GDQDs was somewhat greater than that reported in these studies, the observed fluorescence retention indicates a certain degree of resistance to photobleaching. These findings suggest that GDQDs and M-GDQDs exhibit reasonably good photostability and may have potential for fluorescence imaging and sensing applications. The above results indicate that the prepared quantum dots possess good fluorescence stability and are promising for applications in complex and dynamical environments.

**Figure 4:**
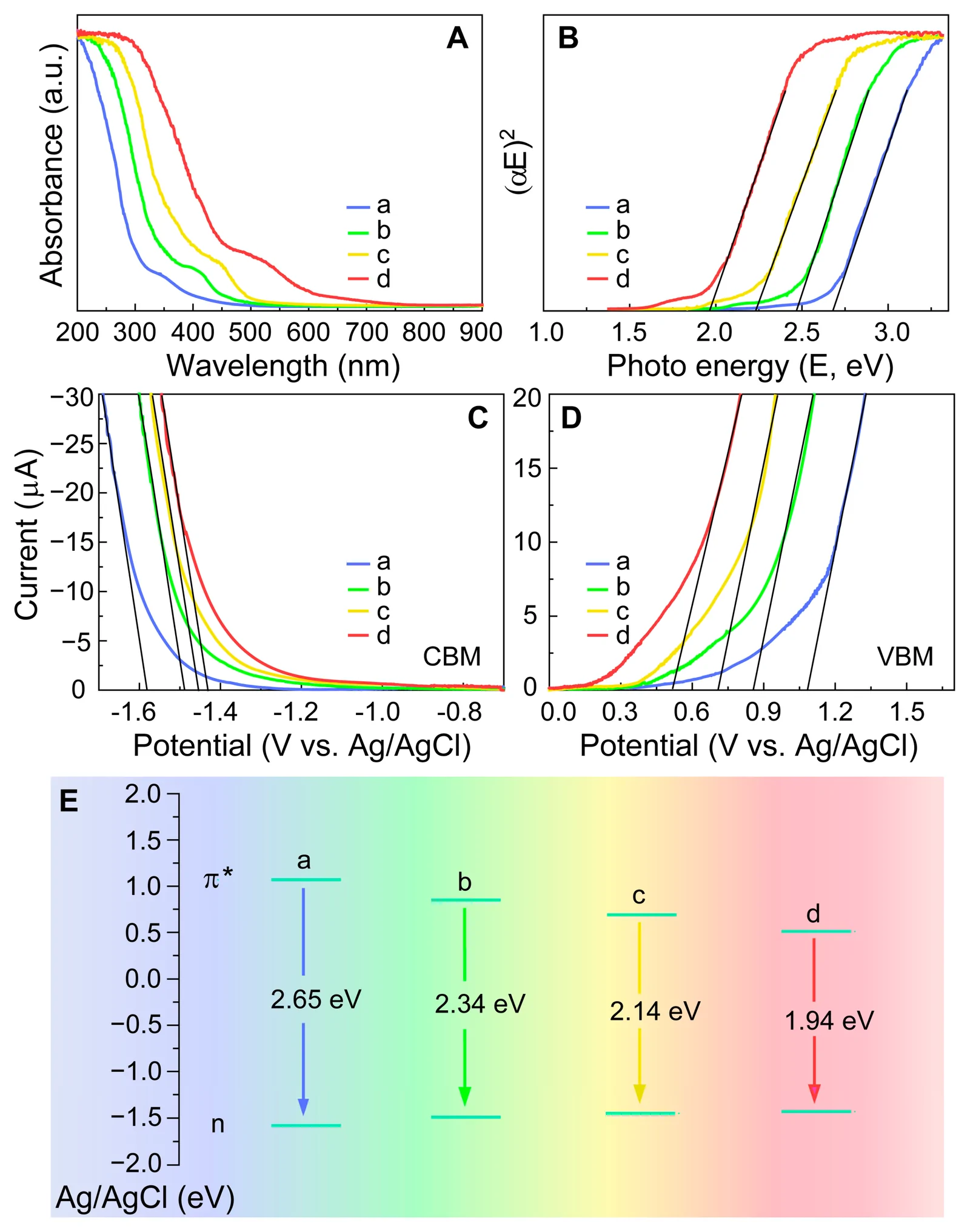
(**A**) UV-Vis-NIR absorption spectra and (**B**) corresponding Tauc plot of (αE)^2^-E, where α is the absorption coefficient, and E represents photon energy. (**C**) Cathodic and (**D**) anodic linear sweep voltammetry at 5 mV s^−1^ to determine CBM and VBM. © Energy level diagrams of several quantum dots. (**E**) Energy level diagrams of several quantum dots, asterisk represents the π antibonding orbital. In all graphs, a, b, c, d represents GDQDs, OPD-GDQDs, D18-GDQDs and BNP-GDQDs, respectively.

To gain a deeper understanding of the reason for the red shift of the PL emission peak, the optical absorption spectrum, linear sweep voltammetry measurements (LSV), and density functional theory (DFT) calculations were performed. Figure 4A reveals that all of these quantum dots display a distinct π-π* transition in the region 200–300 nm, the absorption shoulder appears at approximately 350 nm for GDQDs (blue line a) owing to the electronic transition of n-π* after oxidation treatment [45,46]. And it will extend up to 400 nm, 450 nm, and 530 nm for OPD-GDQDs (green line b), D18-GDQDs (yellow line c), and BNP-GDQDs (red line d) due to the enlargement of the π system and the introduction of nitrogen-containing groups after the condensation of GDQDs and polycyclic aromatic compounds [47,48].

In addition, this enhanced absorption in the visible light range facilitates the emission of quantum dots in the long wavelength range. Then, we plotted the Tauc plot (Figure 4B) from the UV-vis-NIR spectrum above to calculate the HOMO-LUMO gap, and extrapolated the linear region to obtain the x-intercept values which represents the band gap between HUMO and LUMO [49,50]. As Table S9 lists, the values for GDQDs, OPD-GDQDs, D18-GDQDs, and BNP-GDQDs are 2.69 eV, 2.46 eV, 2.20 eV, and 1.96 eV, respectively. And according to the formula Eg = 1240/λ, we could obtain the PL emission wavelengths and observe an increasing tendency, which is consistent with the aforementioned experimental results. Linear sweep voltammetry was used to analyze the conduction band minimum (CBM) and valence band maximum (VBM) (Figure 4C,D) [51], and the difference between CBM and VBM also represents the band gap of these quantum dots. As displayed by the energy diagram in Figure 4E, the CBM (π*) decreases visibly from blue to red quantum dots, whereas the VBM (n) increases mildly. The band gaps determined from this difference were 2.65 eV, 2.34 eV, 2.14 eV, and 1.94 eV, which correspond to GDQDs, OPD-GDQDs, D18-GDQDs, and BNP-GDQDs, respectively. And the reduction in the band gap is in agreement with the UV-vis-NIR spectrum results. These results suggested that the redshift of the absorption spectrum and the reduction in the band gap due to the π-system expansion and electronic structure change does contribute to the regulation of the fluorescence properties.

**Figure 5:**
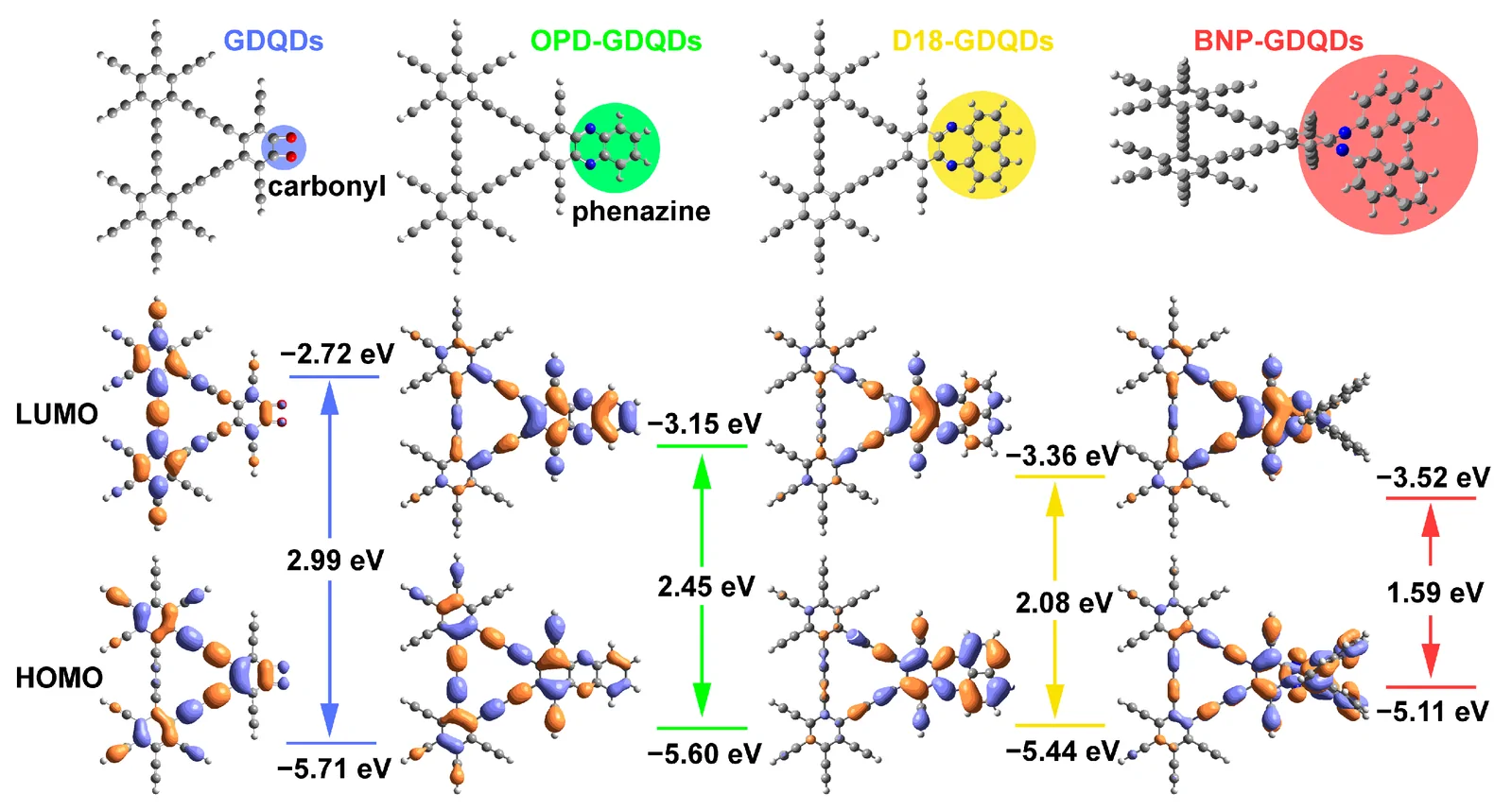
HOMO and LUMO theoretical calculation results for several quantum dots. Take the M-GDQDs with phenazine structure as an example, which is formed by the conjugation between carbonyl group of GDQDs and different polycyclic aromatic amines.

To verify the effect of the π-system enlargement and electronic structure change on the band gap, density functional theoretical calculations were employed to simulate the synthesized quantum dots. To be as close as possible to the experimental results, we built a model close to the actual particle size of GDQDs, as shown in Figure S8, consisting of three graphdiyne monomers without any other functional groups. However, the GDQDs obtained after hydrothermal treatment contain carbonyl, hydroxyl, carboxyl, and epoxy groups. Here, to simplify the description, we calculated the results after the reaction of GDQDs containing only one type of functional group with OPD, D18, and BNP, respectively. Taking the GDQDs containing two carbonyl groups as an example, as shown in Figure 5, the LUMO and HOMO values are −2.72 eV and −5.71 eV, respectively, and the band gap difference is 2.99 eV. After the condensation reaction, phenazine-structured OPD-GDQDs, D18-GDQDs, and BNP-GDQDs are formed, and as the π system increases, the LUMO decreases significantly while the HOMO rises slowly, resulting in the band gap gradually narrowing. And it could be attributed to the formation of uniform electron cloud density and the enhancement of delocalization, which reduces the coupling between electrons and photons, resulting in the stokes shift of the absorption wavelength, excitation wavelength, and emission wavelength [52,53]. Notably, the optical gaps inferred from absorption and electrochemical measurements are method-dependent effective quantities, whereas the DFT values represent orbital-energy differences for idealized, finite molecular models. Solvent environment, functional-group distributions, edge termination, structural relaxation and the known limitations of approximate DFT orbital energies can account for deviations. These energy gaps should not be converted directly into exact emission wavelengths because emission may involve excited-state relaxation and surface-related states.

The band gap of GDQDs with two hydroxyl groups is 2.94 eV (Figure S9), and the change in band gap after reacting with various polycyclic aromatic amines to form phenazine structure is consistent with GDQDs containing carbonyl group (2.45 eV for OPD-GDQDs, 2.08 eV for D18-GDQDs, 1.59 eV for BNP-GDQDs). Figure S10 shows various conjugating structures resulting from the reaction of carboxyl-containing GDQDs with OPD, D18, and BNP. It demonstrates that their band gap declined from 2.84 eV to 2.51 eV as the π system increased. The formation of benzimidazole-containing quantum dots upon carboxyl interaction with OPD, D18, and BNP is depicted in Figure S11, and the band gap continues to decrease as the π system expands. Different from the mentioned structures with amide bond above, the emergence of benzimidazole structure requires the participation of both amino groups in polycyclic aromatic chemicals, which also causes the benzimidazole structures to exert a stronger influence on the band gap than the amide structures. In addition, Table S10 suggests that the band gap of M-GDQDs decreases with the expansion of the π system when the electronic structures are identical; when the number of benzene rings is similar, the formation of phenazine and benzimidazole structures is more favorable for band gap narrowness and red shift of PL emission peaks. And it also means that the impacts on the band gap of electronic structure are weaker than that of the π system, which is consistent with the outcomes of the FTIR and XPS spectra. All of the aforementioned investigations ascertain that this fluorescence can be tuned from blue to red owing to the narrow band gap caused by the enlargement of the π system and the of electronic structure [54,55]. The molecular structures used for DFT are deliberately simplified representatives of possible local chemical motifs; they are not atomistic reconstructions of the heterogeneous experimental particles. The calculation is therefore used to examine the qualitative trends among the related motifs rather than to claim quantitative prediction of experimental energy gaps.

### 2.3. Biosafety, Cellular Uptake Pathways, and Bioimaging Applications

To assess the biosafety of these four typical quantum dots, cytotoxicity and mitochondrial membrane potential were investigated. First, Figure S12 depicts the MCF-7 cell viability after incubation with GDQD, OPD-GDQDs, D18-GDQDs, and BNP-GDQDs. Over 70% of cells are still alive at concentrations of quantum dots as high as 500 μg/mL, indicating that all quantum dots are non-toxic to living cells and available for bioimaging within a certain concentration range. In addition, the mitochondrial membrane potential of living cells has a polarity, JC-1 is rapidly taken into the mitochondria based on the polarity, and multimers will emerge and emit red fluorescence in the mitochondria under the 525 nm excitation wavelength. However, when the cell undergoes apoptosis, the mitochondrial membrane potential is depolarized, JC-1 is released from the mitochondria, the intensity of red fluorescence is diminished, and it exists as a monomer in the cytoplasm and emits green fluorescence. As shown in Figure S13, a JC-1 assay kit was used to monitor the change in the mitochondria membrane potential. After incubation with 100 μg/mL GDQDs for 4 h, MCF-7 cells remain alive and show red fluorescence (Figure S13(A2)), which is similar to the cells without any treatment (Figure S13(A1)). When cells are stimulated with 0.3% hydrogen peroxide, the red fluorescence degrades and the green fluorescence predominates (Figure S13(A3)). The membrane potential results of another three kinds of quantum dots coincide with GDQDs, as displayed in Figure S13(B1–B3) (OPD-GDQDs), Figure S13(C1–C3) (D18-GDQDs), and Figure S13(D1–D3) (BNP-GDQDs). All of the results affirmed that cells incubated with quantum dots were as polar as untreated cells, demonstrating excellent biocompatibility of the prepared quantum dots and the viability for cell imaging.

Next, we investigated the quantum dots uptake pathways within cells. Before verifying which endocytic pathway brings quantum dots into cells, we explored the effect of energy on quantum dots uptake by cells. 

**Figure 6:**
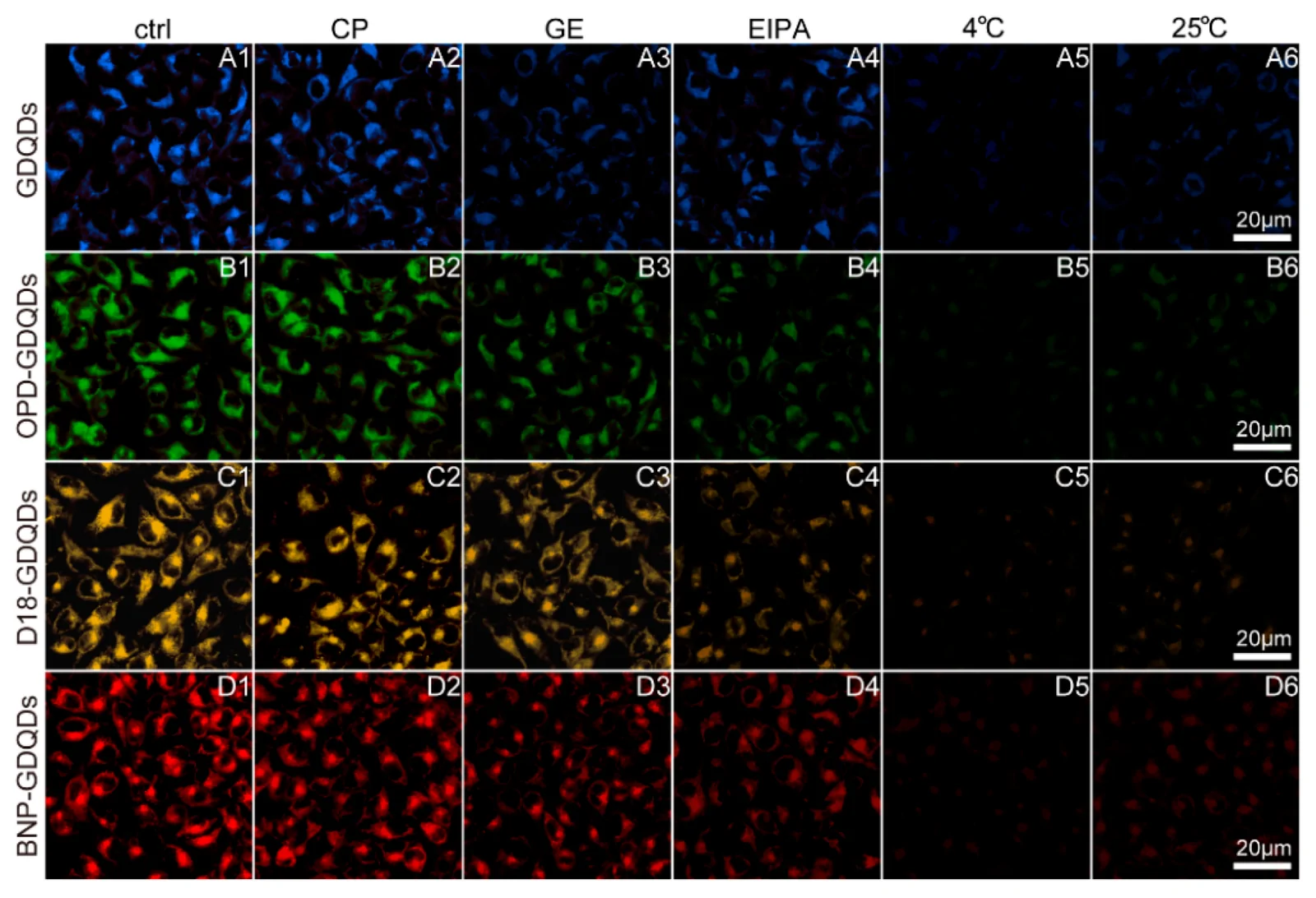
Confocal images of MCF-7 cells incubated with GDQDs (**A1**–**A6**), OPD-GDQDs (**B1**–**B6**), D18-GDQDs (**C1**–**C6**), BNP-GDQDs (**D1**–**D6**) under different conditions, including: in the presence of various endocytosis inhibitors such as chlorpromazine, CP; genistein, GE; 5-(N-ethyl-N-isopropyl) amiloride, EIPA; at different temperature including 4 °C and 25 °C.

The average FL intensity of cells incubated at 4 °C and 25 °C with 100 μg/mL quantum dots is significantly weaker than that at 37 °C, revealing that the ability of cells to take up quantum dots increases with increasing temperature, which involves an energy-dependent endocytosis pathway, and is consistent with previous reports [56,57,58]. To investigate the specific endocytosis pathway, we pretreated these cells with various inhibitors, such as clathrin-mediated endocytosis (inhibitor chlorpromazine, CP), caveolae-mediated endocytosis (inhibitor genistein, GE), and macropinocytosis (inhibitor, 5-(N-ethyl-N-isopropyl) amiloride, EIPA) [59]. According to Figure 6 and Figure S14, there is almost no change in the FL intensity of all quantum dots after incubation with CP, indicating that clathrin-mediated endocytosis is minimally involved in the uptake of all quantum dots. And for GDQDs, the FL intensity dramatically decreases by ~68% in the presence of GE, with minor influence from EIPA pretreatment, indicating that caveolae-mediated endocytosis is the predominant internalization mechanism. However, the FL intensities display a decline of ~73%, 78%, and 70% for OPD-GDQDs, D18-GDQDs, and BNP-GDQDs, respectively, under the effect of EIPA, and little reduction after incubation with GE, suggesting that these three types of quantum dots were primarily taken up by macropinocytosis. We speculate that this distinction between GDQDs and M-GDQDs derives from the formation of conjugating π structures with nitrogen functional groups.

**Figure 7:**
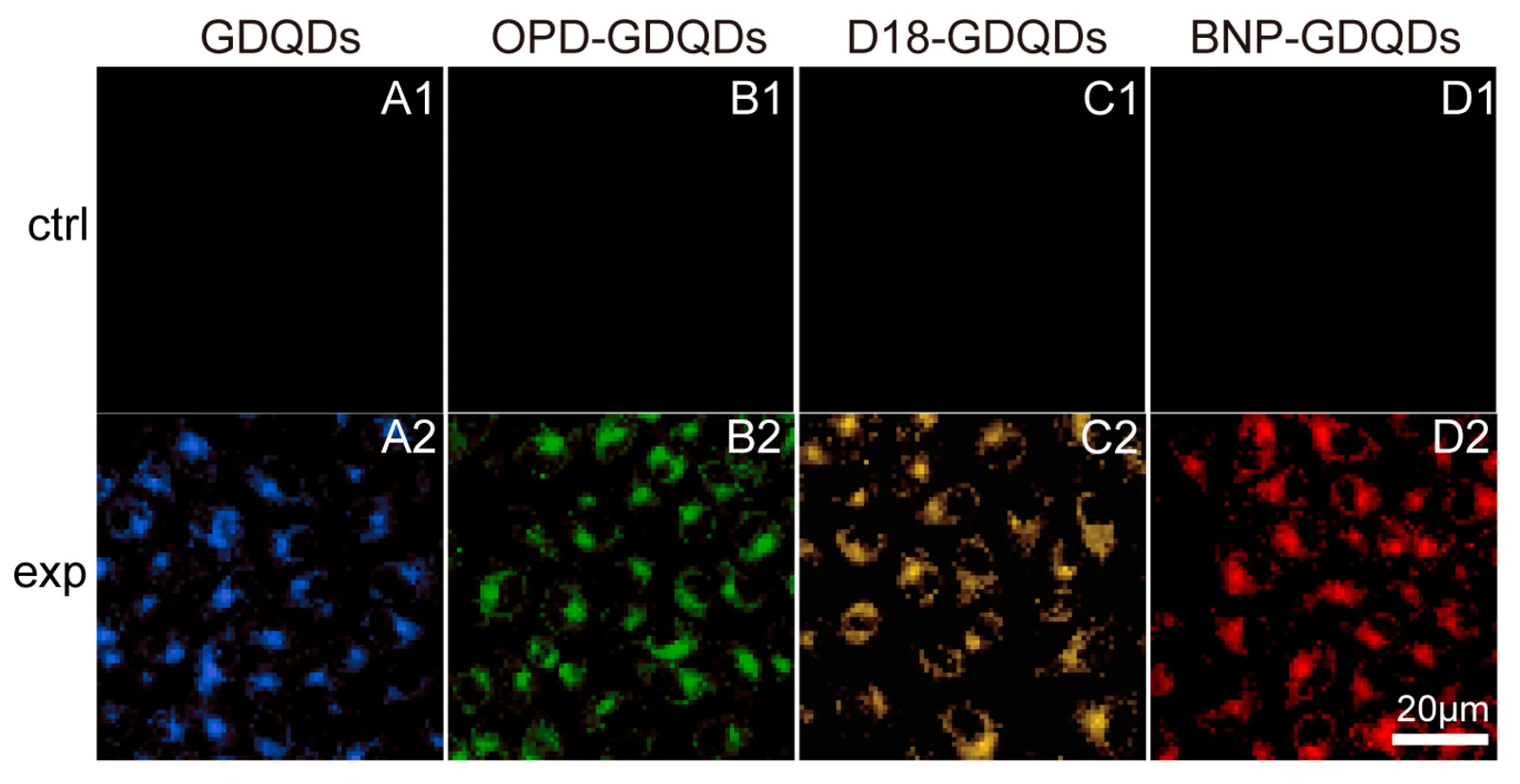
Confocal images of untreated MCF-7 cells (negative controls, (**A1**–**D1**)) and MCF-7 cells incubated with GDQDs (**A2**), OPD-GDQDs (**B2**), D18-GDQDs (**C2**), and BNP-GDQDs (**D2**) at a concentration of 100 μg/mL for 4 h. The excitation wavelengths were 405, 488, 488, and 561 nm for the four quantum dots, respectively.

Finally, the potential of GDQDs and M-GDQDs for cellular fluorescence imaging was evaluated using MCF-7 cells. As shown in Figure 7, untreated MCF-7 cells exhibited negligible background fluorescence signals in the corresponding detection channel Figure 7(A1–D1). In contrast, cells incubated with 100 μg/mL GDQDs, OPD-GDQDs, D18-GDQDs, and BNP-GDQDs for 4 h exhibited distinct blue, green, yellow, and red fluorescence signals, respectively, as shown in Figure 7(A2–D2). These observations suggest that the detected fluorescence primarily originates from the introduced quantum dots rather than intrinsic cellular autofluorescence. The results indicate that these quantum dots not only penetrate the cell membranes of MCF-7 cells without any treatment, but also disperse in the cytoplasm via various endocytosis pathways [60,61]. Furthermore, the fluorescence signals observed in the treated cells, together with the cellular uptake results discussed above, support the potential application of GDQDs and M-GDQDs as multicolor fluorescence imaging probes. Overall, these findings demonstrate the feasibility of using the prepared GDQDs and M-GDQDs for multicolor cellular fluorescence imaging.

## 3. Materials and Methods

### 3.1. Materials and Reagents

GDY was synthesized according to our previous work. Sinopharm Chemical Reagent Co. (Shanghai, China) supplied 30% H_2_O_2_, ethanol, o-phenylenediamine (OPD), 1,8-diaminonaphthalene (D18), 1,1′-bi(2-naphthylamine) (BNP). Dulbecco’s modified Eagle medium (DMEM) supplemented with fetal bovine serum (FBS, 10%), L-glutamine (2 mM), penicillin (100 U mL^−1^), and streptomycin, 3-(4,5-dimethyldiazol-2-yl)-2,5-diphenyltetrazolium bromide (MTT), chlorpromazine(CP), genistein, 5-(N-ethyl-N-isopropyl)amiloride (EIPA) were supplied by Sangon Biotechnology Company, Ltd. (Shanghai, China). The mitochondrial membrane potential assay kit with JC-1 was purchased from Beyotime (Shanghai, China). All chemicals were commercially available, of analytical grade, and were used as received without any further purification. All aqueous solutions were prepared using deionized (DI) water with a resistivity of approximately 18.2 MΩ cm. A dialysis bag (MWCO 500 Da) was purchased from Yuanye Biotechnology Co. Ltd. (Shanghai, China).

### 3.2. Characterization

Transmission electron microscope (TEM) images of the synthesized GDQDs were recorded on a JEOL JEM-2100F TEM (JEOL Ltd., Akishima, Japan) operating at an accelerating voltage of 200 kV. The TEM samples were prepared by drying a droplet of the GDQDs solution on a carbon film-support grid. Particle-size distributions reported in this article were determined from TEM micrographs of dried specimens. XRD patterns of the GDQDs were recorded on a Rigaku/Max-3A X-ray diffractometer (Rigaku Corporation, Tokyo, Japan) with Cu Kα radiation (λ = 0.15418 nm). The Raman spectra were recorded on a Labram HR 800 micro spectrometer (HORIBA Jobin Yvon, Longjumeau, France) with an excitation source of 532 nm. The FTIR transmission spectra were recorded at a resolution of 4 cm^−1^ using a Nexus 670 FTIR spectrophotometer (Nicolet Instruments, Madison, WI, USA) for samples on a KBr disk. The surface characteristics of the GDQDs were examined by X-ray photoelectron spectroscopy (XPS), which was measured with an ESCALAB 250 XPS spectrometer (VG Scientific, East Grinstead, UK) using a monochromatic Al Kα line at 1486.6 eV. Binding energies were calibrated concerning the C 1s peak at 284.6 eV. Peak fit analysis was performed using the XPS PEAK program (V4.0). The UV−vis−NIR spectra were measured by a Cary 5000 UV−vis−NIR spectrometer (Varian Inc., Palo Alto, CA, USA). Fluorescence and the photoluminescence excitation (PLE) spectra were measured using a fluoroSENS fluorescence spectrophotometer (Gilden Photonics Ltd., Clydebank, UK) equipped with a xenon lamp excitation source (150 w). The fluorescence decays were collected by a Fluoro-Max-4 Spectrofluorometer (FM-4P-TCSPC, with standard configuration, HORIBA Jobin Yvon, Longjumeau, France) using an RR928P-2 detector. The lifetime data were obtained by the application of deconvolution techniques using the IBH DAS6 analysis software (version 6.4, HORIBA Scientific). A Fluoro-Max-4 Spectrofluorometer (FM-4P-TCSPC, HORIBA Jobin Yvon) equipped with an integrating sphere was employed to obtain the absolute QYs.

### 3.3. Synthesis of GDQDs and M-GDQDs

GDQDs were obtained from GDY by using the hydrothermal method. Specifically, 12 mg of GDY was dispersed in a mixture of ddH_2_O (21 mL) and 30% hydrogen peroxide (3 mL) (3 mL 30% hydrogen peroxide was mixed with 12 mL of 1 mg/mL GDY aqueous solution first and then the mixture was diluted with 9 mL deionized water). Next, the mixture was ultra-sonicated for 2 h then transferred into a 50 mL Teflon-lined autoclave and heated to 180 °C for 12 h [62]. After cooling to room temperature, the suspension was filtered through 0.22 μm microporous membrane and then dialyzed with a dialysis bag (MWCO 500 Da) for 48 h to obtain freeze-dried GDQDs. The procedure for preparing modified GDQDs (M-GDQDs) is as follows [24,32]: 30 mg *o*-phenylenediamine (OPD), 1,8-diaminonaphthalene (D18), 1,1′-bi(2-naphthalene) (BNP) was added in the 6 mL GDQDs ethanol solution (1 mg/mL) and then each was diluted with 9 mL of ddH_2_O, respectively. After ultrasonicating for 30 min, the three mixtures were reacted in a sealed Teflon-lined stainless-steel autoclave at 180 °C for 12 h, respectively. When the hydrothermal reaction was finished, all of the autoclaves were naturally cooled down to room temperature. The resultant suspensions were centrifuged with Mili-Q water three times at 11,000 rpm for 10 min to collect the black precipitants, followed by redispersing three precipitants into water under sonication to form a homogeneous brown-blackish aqueous solution for dialysis with a molecular weight cutoff of 500 Da for 3 days. Subsequently, three kinds of modified GDQDs were obtained by freeze-drying and stored for further characterization.

### 3.4. Quantum Yields (QYs) Measurement

The absolute QYs were tested using an integrating sphere attached to an FM-4P-TCSPC spectrofluorometer (HORIBA Jobin Yvon, Longjumeau, France) [63]. And the QY was determined by the ratio between photons emitted and absorbed by M-GDQDs. First, the solution of prepared GDQDs was diluted to an absorbance of below 0.1 at an optimal PLE wavelength of 380 nm. Subsequently, the solution was added to a 10 mm fluorescence cuvette, placed in the integrating sphere, and excited with the optical wavelength; the PL emission spectra were recorded in the range of 370–390 and 395–730 nm. The PL spectra of pure water were also recorded under identical conditions. Finally, the QYs were calculated from the spectra of both the sample and the water by using fluorescence software. Each experiment was performed three times in parallel to obtain the average QY value. The QYs of other quantum dots were also obtained by the identical method with different excitation wavelengths and range of PL emission wavelengths.

### 3.5. Theoretical Calculations

Electronic structure calculations of GDQDs and the three kinds of M-GDQDs with various terminal groups were conducted using the Gaussian 09 program package ((Gaussian, Inc., Wallingford, CT, USA)). The density functional theory (DFT) computations were performed using the B3LYP functional and 6-31 + G(d,p) basis sets. To simplify the calculation without a loss of generality, the model of GDQDs used here is the one with only three graphdiyne monomers. And the modified quantum dots just contain one conjugated moiety, which is formed by combining the functional groups (carbonyl, carboxyl, hydroxyl) of GDQDs with three kinds of chemicals (OPD, D18, BNP), respectively. The carbon atoms at the edge of each model were terminated with hydrogen atoms. All geometrical structures were optimized using the DFT method with a B3LYP functional. Then we calculated the energy of HOMO and LUMO, and the band gap was calculated by subtracting the HOMO from the LUMO. Furthermore, the spatial distributions of electrons in HOMO and LUMO for GDQDs, OPD-GDQDs, D18-GDQDs, and BNP-GDQDs were also evaluated from the calculated results. The calculations used the B3LYP/6-31 + G(d,p) level as stated above. No solvent model or D3 dispersion correction was specified in the original computational protocol.

### 3.6. Investigation of Cellular Internalization Pathway and Cellular Imaging

First, the MCF-7 cells were seeded in confocal Petri dishes at a density of 5 × 10^3^ cells/mL in a medium and cultured for 12 h. To assess the energy-dependency of cellular uptake of quantum dots, the MCF-7 cells were incubated with the four types of quantum dots at 100 μg/mL for 4 h at 4 °C and 25 °C, respectively. Then, the cells were washed with PBS and fluorescence imaged on a confocal laser scanning microscope (CLSM, MRC-1024, Bio-Rad, Ltd., USA). And for the investigation of cellular internalization pathway of GDQDs and M-GDQDs, the cells were preincubated with fresh medium for 2 h at 37 °C containing different pathway inhibitors: 30 μM chlorpromazine (CP) for clathrin-mediated endocytosis, 100 μM genistein (GE) for caveolae-mediated endocytosis, and 100 μM 5-(N-ethyl-N-isopropyl) amiloride (EIPA) for micropinocytosis. Then the medium was replaced with fresh DMEM containing 100 μg/mL quantum dots for 4 h and fluorescence was observed on CLSM. The MCF-7 cells directly treated with all kinds of 100 μg/mL quantum dots at 37 °C for 4 h were used as control. For cellular imaging, the MCF-7 cells (1 × 10^4^ cells per confocal dish) were cultured for 24 h at 37 °C in a humidified atmosphere containing 5% CO_2_. Subsequently, the cells were incubated with GDQDs, OPD-GDQDs, D18-GDQDs, or BNP-GDQDs at a concentration of 100 μg/mL for 4 h. After incubation, the cells were washed three times with phosphate-buffered saline (PBS) to remove excess quantum dots. Untreated MCF-7 cells were included as negative controls to evaluate cellular autofluorescence. The excitation wavelengths used for GDQDs, OPD-GDQDs, D18-GDQDs, and BNP-GDQDs were 405, 488, 488, and 561 nm, respectively. The recorded laser power settings were 7.18% at 405 nm, 6.55% at 488 nm, and 8.76% at 561 nm. The images were acquired at a nominal scan rate of 0.25 frames/s, corresponding to approximately 4 s per frame, with an image resolution of 1024 × 1024 pixels and without line averaging.

## 4. Conclusions

In summary, we accomplished tunable fluorescence of GDQDs via conventional hydrothermal and solvothermal treatment. Optical measurements and representative theoretical calculations are consistent with the contribution from conjugation-related changes and altered electronic environments to the observed red shift, although the individual effects cannot be quantitatively isolated from the current dataset. After modification, we obtained OPD-GDQDs with green fluorescence, D18-GDQDs with yellow fluorescence, and BNP-GDQDs with red fluorescence. In addition, their advisable optical properties, fine solubility in water, and biocompatibility render them capable of bioimaging. The work extends the reported range of fluorescence colors for the GDQD-derived materials investigated here. And although they do not possess the high QYs and long fluorescence lifetime like other CNPs, we believe that they will be beneficial for applications in energy, catalysis, optoelectronic devices, biosensors, nanomedicine, and other related fields.

## Data Availability

Data are contained within the article and Supplementary Materials.

## Supplementary Materials

The following supporting information can be downloaded at: https://www.mdpi.com/article/10.3390/molecules31213998/s1. Table S1: The values of ID/IG of GDQDs and M-GDQDs; Table S2: The percentage of elements (C, N, O) in the four types of quantum dots; Table S3: Ratios of atoms in the four kinds of quantum dots; Table S4: Percentage of different bonding types of carbon; Table S5: The integrated area ratios of sp2 C/sp C for various quantum dots; Table S6: Percentage of different bonding types of nitrogen; Table S7: Percentage of different bonding types of oxygen; Table S8: Fluorescence lifetime and absolute quantum yields of various quantum dots; Table S9: Band gap values calculated from absorption spectrum; Table S10: Effects on band gap of electronic structures and effective conjugation length; Figure S1: XRD patterns of GDQDs, OPD-GDQDs, D18-GDQDs, BNP-GDQDs; Figure S2: PL spectra of GDQDs and M-GDQDs under different λex; Figure S3: Normalized PL emission spectrum of GDQDs and M-GDQDs; Figure S4: Maximum PLE and PL emission spectrum of GDQDs and M-GDQDs; Figure S5: Normalized PL intensity of GDQDs and M-GDQDs at different pH values; Figure S6: Effect of ionic strengths on the PL intensity of GDQDs and M-GDQDs; Figure S7: PL stability of GDQDs and M-GDQDs; Figure S8: Theoretical simulation results of graphdiyne model with three monomers; Figure S9: Theoretical simulation results of M-GDQDs with phenazine structures; Figure S10: Theoretical simulation results of M-GDQDs with amide structures; Figure S11: Theoretical simulation results of M-GDQDs with benzoimidazole structures; Figure S12: Cytotoxicity of GDQDs and M-GDQDs; Figure S13: Mitochondrial membrane potential of MCF-7 cells; Figure S14: Mean fluorescence intensities of MCF-7 cells under different conditions.
