# Supplementary material for: Tunable Fluorescence of Graphdiyne Quantum Dots by Enlarging π System and Altering Electronic Structure
Source: Molecules. 2026 Nov 6;31(21):3998. doi: 10.3390/molecules31213998 (PMC13764890; doi:10.3390/molecules31213998)
Supplement: Supplementary file 1 [file molecules-31-03998-s001.zip › molecules-4657071-supplementary.pdf]

Supporting Information:

Tunable fluorescence of graphdiyne quantum dots by enlarging π system and altering electronic structure

Ling Qiao[1], Wenchao Lang[2], Jia Luo[1], Xiaoqing Xu[1,*]

[1]School of Pharmacy and Laboratory Medicine, Jiangsu Health Vocational College, Nanjing 211800, P. R. China. qiaoling0516@126.com(L.Q.);

[2]School of Chemistry, Chemical Engineering & Biotechnology, Nanyang Technological University, Singapore 637371, Singapore.

*Correspondence: xuxiaoqing122@163.com(X.X.)

CONTENTS:

1. Experimental

2. Tables

Table S1. The values of I_D/I_G of GDQDs and M-GDQDs.

Table S2. The percentage of elements (C, N, O) in the four types of quantum dots.

Table S3. Ratios of atoms in the four kinds of quantum dots.

Table S4. Percentage of different bonding types of carbon.

Table S5. The integrated area ratios of sp2 C/sp C for various quantum dots.

Table S6. Percentage of different bonding types of nitrogen.

Table S7. Percentage of different bonding types of oxygen.

Table S8. Fluorescence lifetime and absolute quantum yields of various quantum dots.

Table S9. Band gap values calculated from absorption spectrum.

Table S10. Effects on band gap of electronic structures and effective conjugation length.

3. Figures

Figure S1. XRD patterns of GDQDs, OPD-GDQDs, D18-GDQDs, BNP-GDQDs.

Figure S2. PL spectra of GDQDs and M-GDQDs under different λex.

Figure S3. Normalized PL emission spectrum of GDQDs and M-GDQDs.

Figure S4. Maximum PLE and PL emission spectrum of GDQDs and M-GDQDs.

Figure S5. Normalized PL intensity of GDQDs and M-GDQDs at different pH values.

Figure S6. Effect of ionic strengths on the PL intensity of GDQDs and M-GDQDs.

Figure S7. PL stability of GDQDs and M-GDQDs.

Figure S8. Theoretical simulation results of graphdiyne model with three monomers.

Figure S9. Theoretical simulation results of M-GDQDs with phenazine structures.

Figure S10. Theoretical simulation results of M-GDQDs with amide structures.

Figure S11. Theoretical simulation results of M-GDQDs with benzoimidazole structures.

Figure S12. Cytotoxicity of GDQDs and M-GDQDs.

Figure S13. Mitochondrial membrane potential of MCF-7 cells.

Figure S14. Mean fluorescence intensities of MCF-7 cells under different conditions.

1. Experimental

Cell Culture. MCF-7 (human breast cancer cell) cell line was obtained from the cell bank of the type culture collection of the Chinese Academy of Sciences (Shanghai, China) and cultured at 37 °C in Dulbecco's modified Eagle medium (DMEM) supplemented with fetal bovine serum (FBS, 10%), L-glutamine (2 mM), penicillin (100 U mL^{-1}), and streptomycin (100 μg mL^{-1}) in a CO_2 environment (5%). After growing to 90% confluence, the cells were washed with PBS, and the culture medium was replaced by 1 mL PBS. The cell number was estimated using a hemocytometer.

Measurement of Mitochondrial Membrane Potential. MCF-7 cells were seeded in 35 mm Petri dishes at a density of 5×10^3 cells/mL for 12 h and then incubated with GDQDs and M-GDQDs for 4 h. Cells were treated with 0.3% H_2O_2 diluted in medium for 20 min as a positive control. After being washed with PBS three times, cells were stained with JC-1 (10 μg/mL) at 37 °C for 20 min. Then cells were washed with PBS three times for imaging on confocal laser scanning microscope (CLSM, MRC-1024, Bio-Rad, Ltd., USA).

2. Tables

Table S1. The values of I_D/I_G of GDQDs, OPD-GDQDs, D18-GDQDs, BNP-GDQDs.

Type	I_D/I_G
GDQDs	0.98
OPD-GDQDs	0.96
D18-GDQDs	0.93
BNP-GDQDs	0.9

Tables S2. The percentage of elements (C, N, O) in the four types of quantum dots.

Type	C (%)	O (%)	N (%)
GDQDs	72.73	27.27	0
OPD-GDQDs	75.79	21.38	2.83
D18-GDQDs	77.76	19.60	2.64
BNP-GDQDs	79.52	18.42	2.06

Table S3. Ratios of atoms in the four kinds of quantum dots.

Type	C/O	N/C
GDQDs	2.68	0
OPD-GDQDs	3.54	0.037
D18-GDQDs	3.97	0.034
BNP-GDQDs	4.32	0.026

Table S4. Percentage of different bonding types of carbon from C 1s XPS.

Type	C=C (%)	C≡C (%)	C=N (%)	C-O/C-N (%)	C=O (%)
GDQDs	36	34	0	16	14
OPD-GDQDs	40	32	13	10	5
D18-GDQDs	43	33	12	8	4
BNP-GDQDs	48	33	9	7	3

Table S5. The integrated area ratios of sp^2 C/sp C for various quantum dots.

Type	area ratio of sp^2 C/sp C
GDQDs	1.06
OPD-GDQDs	1.25
D18-GDQDs	1.30
BNP-GDQDs	1.45

Table S6. Percentage of different bonding types of nitrogen from N 1s XPS.

Type	phenazine N (%)	amino/pyrrolic N (%)	amide/quaternary N (%)
OPD-GDQDs	63	18	19
D18-GDQDs	51	25	24
BNP-GDQDs	56	22	22

Table S7. Percentage of different bonding types of oxygen from O 1s XPS.

**Type**	**O=C-OH (%)**	**C-O (%)**	**C=O (%)**
GDQDs	11	43	46
OPD-GDQDs	9	52	39
D18-GDQDs	6	66	28
BNP-GDQDS	4	87	9

Table S8. Fluorescence lifetime and absolute quantum yields of various quantum dots.

**Type**	**τ_{Av}(ns)**	**QYs(%)**	**k_r (ns^{-1})**	**K_{nr} (ns^{-1})**
GDQDs	2.57	14.96	0.0582	0.3309
OPD-GDQDs	2.50	13.57	0.0543	0.3457
D18-GDQDs	1.95	10.01	0.0513	0.4615
BNP-GDQDs	1.23	7.41	0.0602	0.7528

Table S9. The band gap values and PL emission wavelength calculated from absorptiospectrum of all quantum dots.

**Type**	**Band gap(eV)**	**λ/nm**
GDQDs	2.69	461
OPD-GDQDs	2.46	504
D18-GDQDs	2.20	564
BNP-GDQDs	1.96	633

Table S10. The effects on band gap of electronic structure and π system.

Type	C=O/-OH (phenazine)	-COOH (amide)	-COOH (benzoimidazole)
OPD-GDQDs	2.45	2.84	2.41
D18-GDQDs	2.08	2.56	2.03
BNP-GDQDs	1.59	2.51	1.58

3. Figures

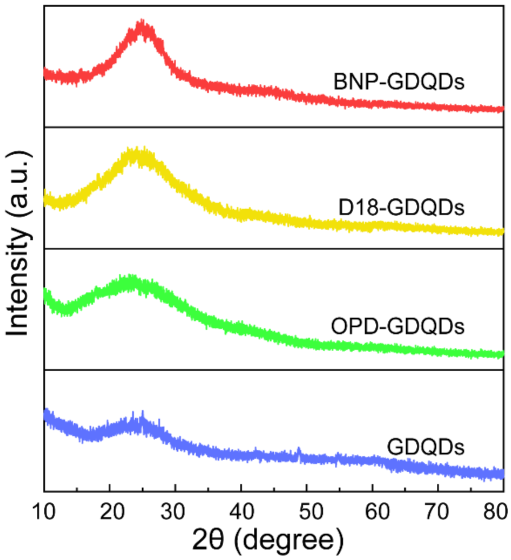

Figure S1. XRD patterns of GDQDs, OPD-GDQDs, D18-GDQDs, BNP-GDQDs.

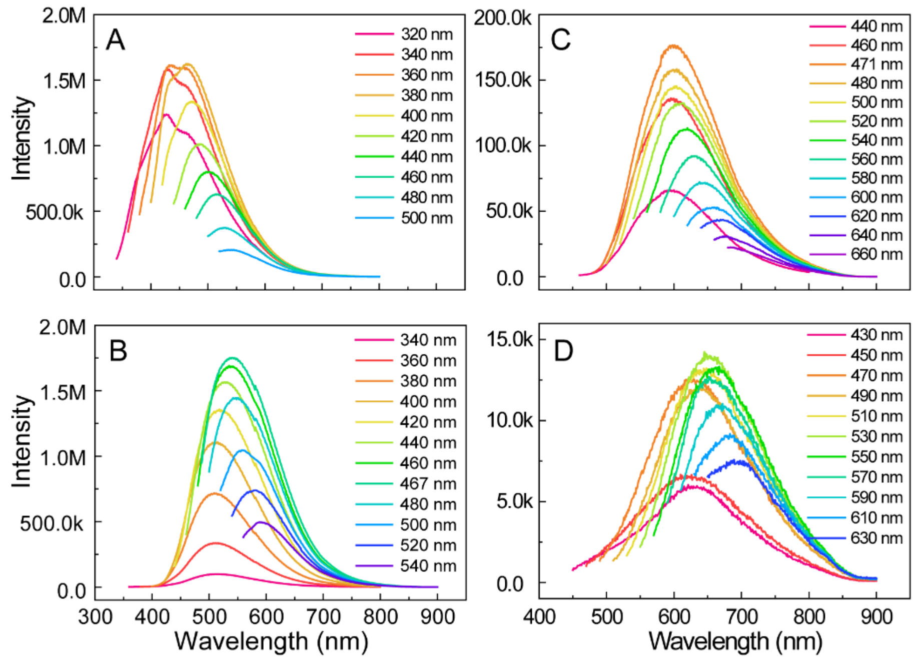

Figure S2. PL spectra of GDQDs(A), OPD-GDQDs(B), D18-GDQDs(C), BNP-GDQDs(D).

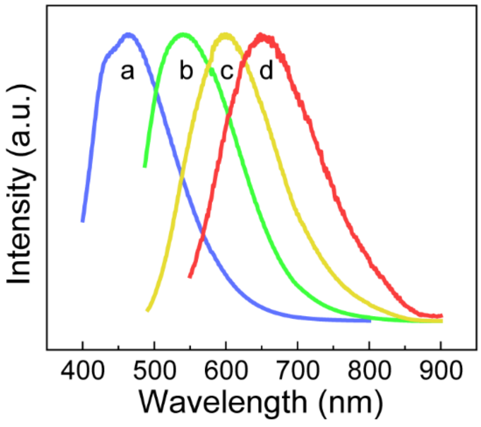

Figure S3. Normalized PL emission spectrum of GDQDs(line a), OPD-GDQDs(line b), D18-GDQDs(line c), BNP-GDQDs(line d).

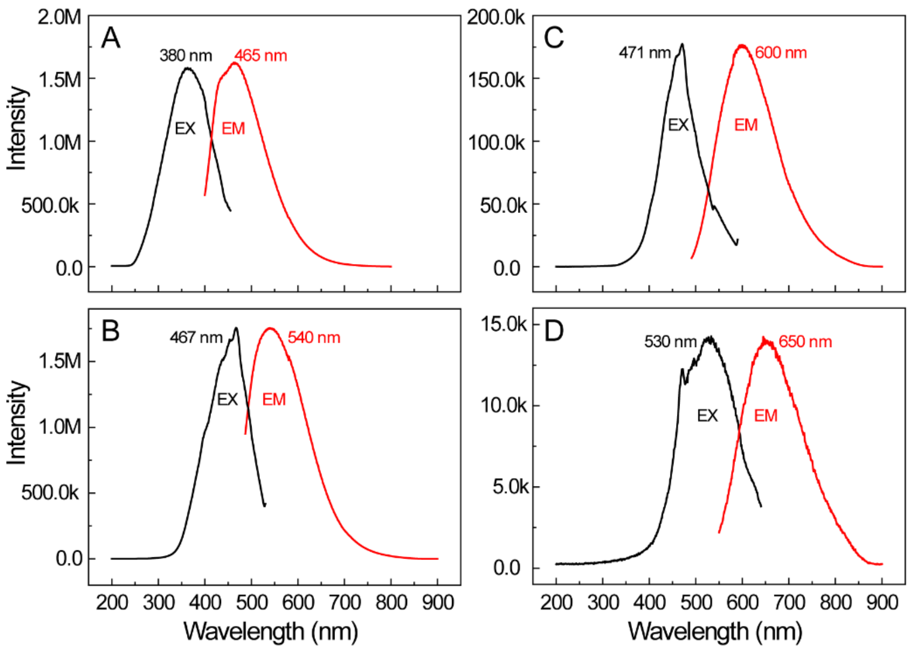

Figure S4. Maximum PLE excitation spectrum of GDQDs(A), OPD-GDQDs(B), D18-GDQDs(C), BNP-GDQDs(D) under the best PL emission wavelength.

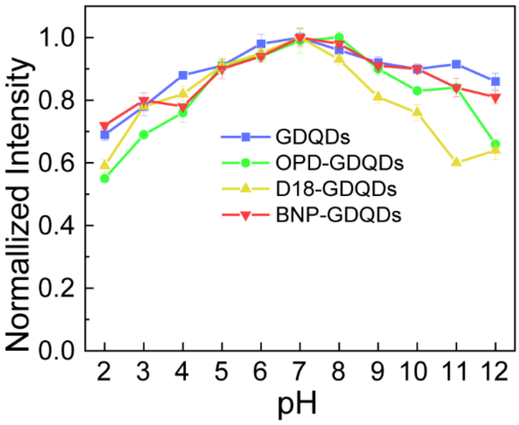

Figure S5. Normalized fluorescence intensity of the four quantum dots at different pH values (2-12).

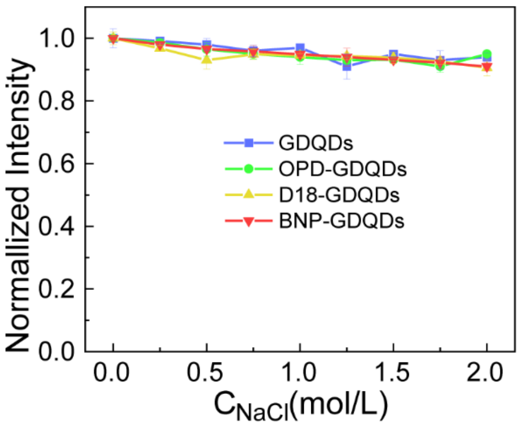

Figure S6. Effect of ionic strengths on the PL intensity of four quantum dots (ionic strengths were controlled by various concentrations of NaCl).

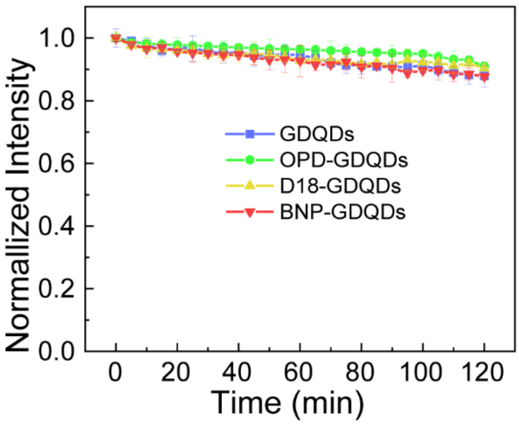

Figure S7. PL intensity changes of quantum dots under 2 hours of continuous irradiation excited by respective maximum PLE wavelength.

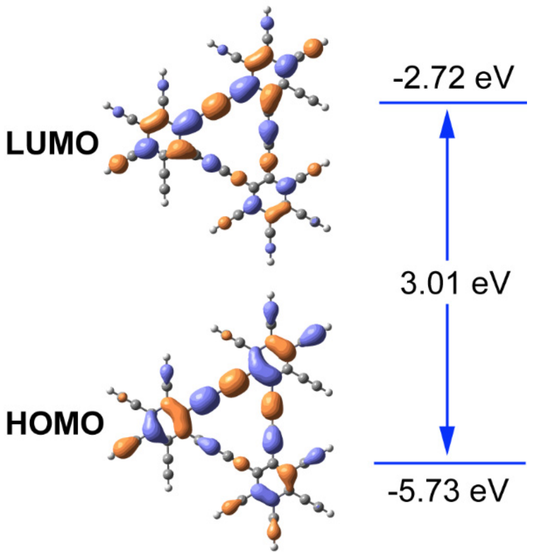

Figure S8. HOMO and LUMO theoretical calculation results of graphdiyne model formed by three monomers.

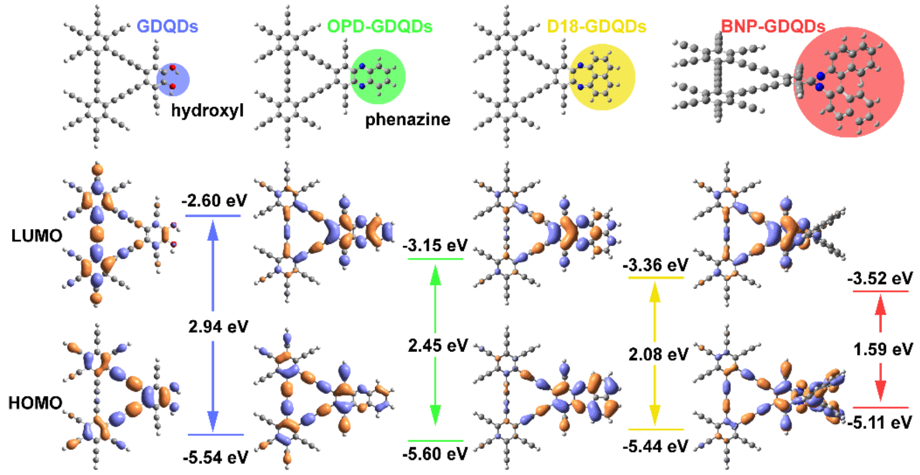

Figure S9. HOMO and LUMO theoretical calculation results for several quantum dots. Take the M-GDQDs with phenazine structure as an example, which is formed by hydroxyl group of GDQDs conjugated with different polycyclic aromatic amines.

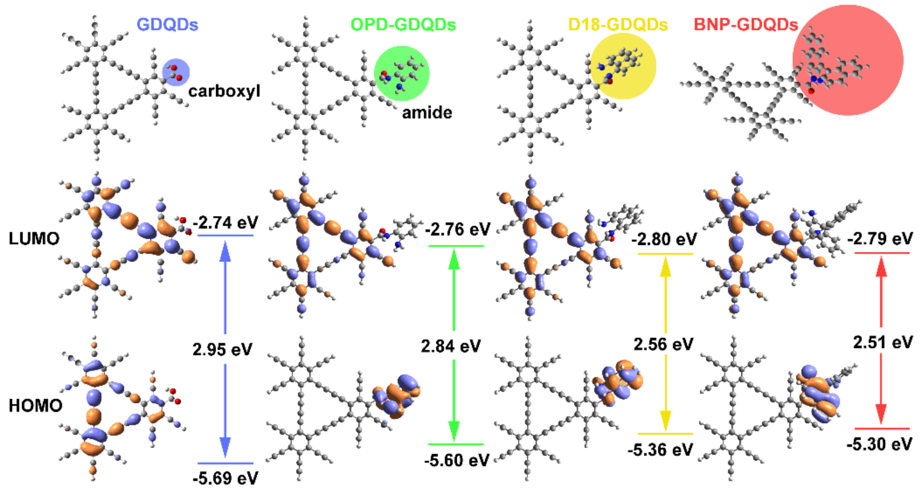

Figure S10. HOMO and LUMO theoretical calculation results for several quantum dots. Take the M-GDQDs with amide structure as an example, which is formed by carboxyl group of GDQDs conjugated with different polycyclic aromatic amines.

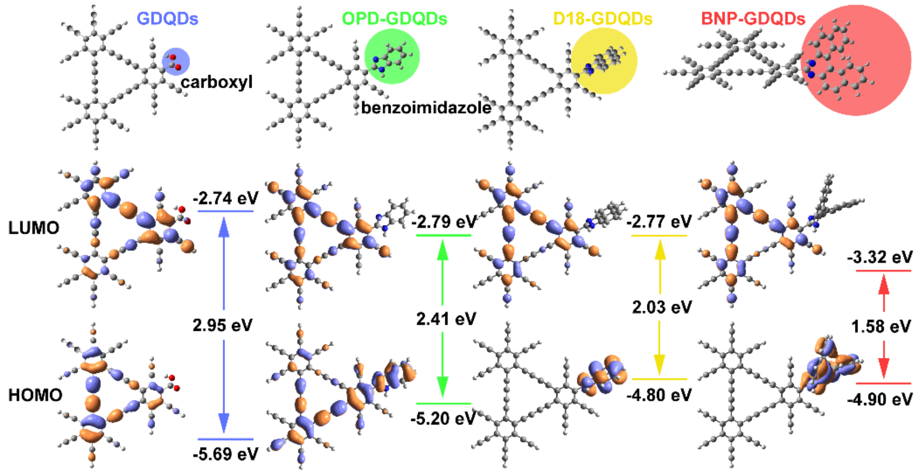

Figure S11. HOMO and LUMO theoretical calculation results for several quantum dots. Take the M-GDQDs with benzimidazole structure as an example, which is formed by the reaction between carboxyl group of GDQDs and different polycyclic aromatic amines.

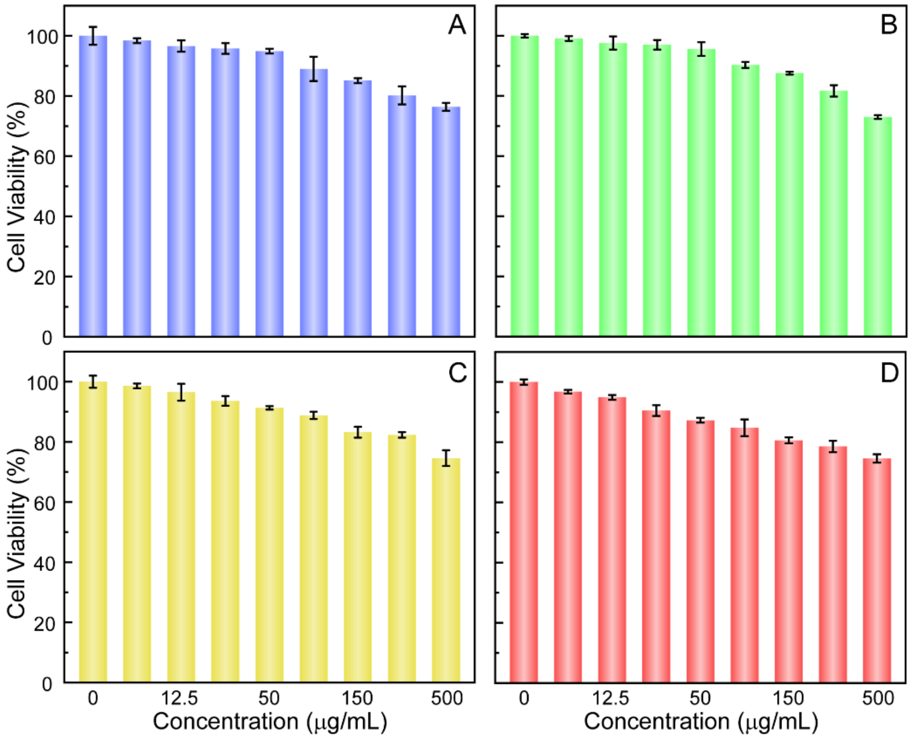

Figure S12. Cell viability after incubated with GDQDs(A), OPD-GDQDs(B), D18-GDQDs(C), BNP-GDQDs(D) at different concentrations. Three independent biological experiments were conducted on different days, with three technical replicates (parallel wells) per experimental condition in each experiment. Data are presented as mean ± standard deviation (SD) calculated from the mean values of the three independent experiments (n = 3).

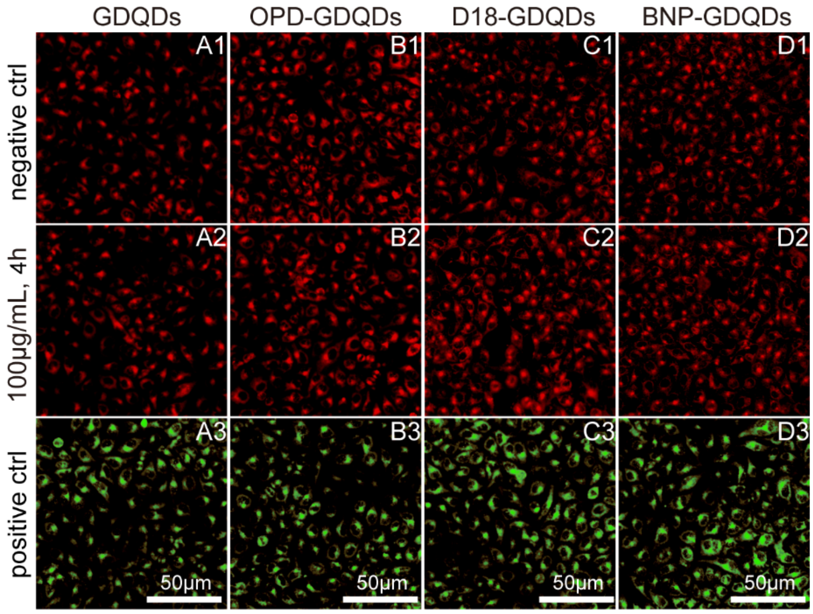

Figure S13. Mitochondrial membrane potential investigation. CLSM images of MCF-7 cells after incubated with various quantum dots for 4 h. Cells treated with H_2O_2 were used as positive control.

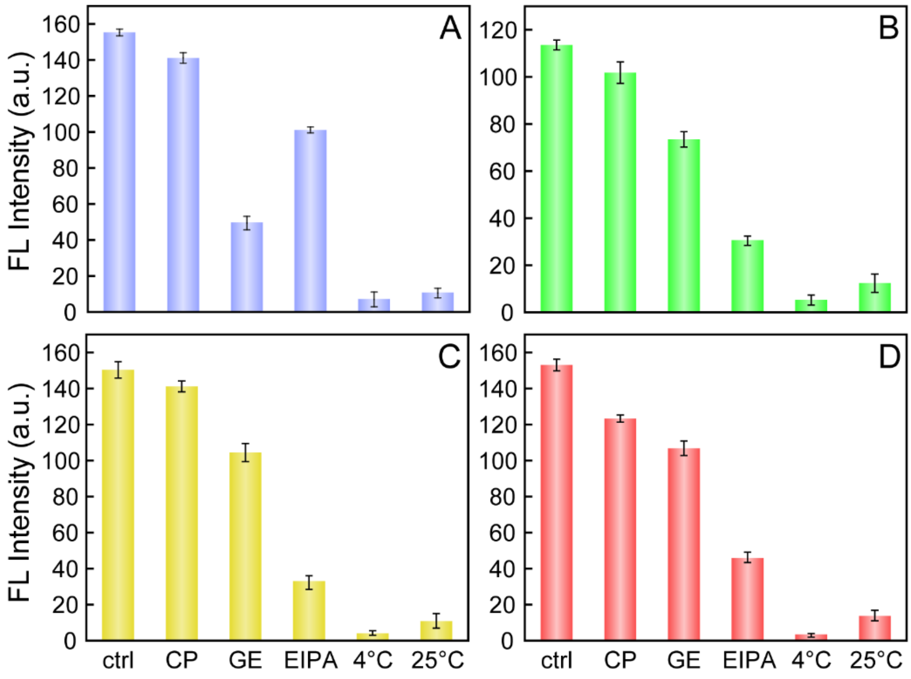

Figure S14. Mean fluorescence intensities of MCF-7 cells under various conditions after incubated with GDQDs(A), OPD-GDQDs(B), D18-GDQDs(C), BNP-GDQDs(D). Fluorescence intensities of multiple cells selected from confocal microscopy images

were quantified using ImageJ software. Error bars represent the standard deviation (SD) of the measured cellular fluorescence intensities.
